# Baseline Stroke Literacy of Young Children Based on “FAST 112 Heroes” Program

**DOI:** 10.3389/fpubh.2021.638516

**Published:** 2021-05-14

**Authors:** Kalliopi Tsakpounidou, Socrates Psomiadis, Tatiana Pourliaka, Maria Akritidou, Hariklia Proios

**Affiliations:** ^1^Department of Educational and Social Policy, University of Macedonia, Thessaloniki, Greece; ^2^Anatolia College, Thessaloniki, Greece

**Keywords:** children, baseline knowledge, health literacy, preschoolers, stroke, stroke awareness, stroke knowledge

## Abstract

**Background:** Young children are often unaware of emergency health conditions, such as stroke, and could serve as important vehicles to save the lives of their grandparents, who are more likely to suffer a stroke. An important aspect for the evaluation of public awareness on stroke signs and related emergency procedures is to examine the level of baseline stroke knowledge children have and whether they understand when to seek medical care on time.

**Objective:** To examine the level of stroke symptomatology knowledge in children as well as evaluate their preparedness in stroke response before their participation in the educational program “FAST (Face, Arms, Speech, Time) 112 Heroes.”

**Methods:** For the purpose of this work, a questionnaire was developed and adapted to preschoolers' needs. The present study involved 123 children (65 boys, 58 girls, aged 4–6.5 years; mean age: 5.30, S.D.: 0.59) from two cities in Greece. Five multiple-choice animated pictures, that were age-appropriate, were administrated to each child, along with verbal explanations provided by the investigator.

**Results:** More than half of the participants (*n* = 65, 52.8%) could recognize the symptom of face drooping, 53 children (43.1%) could identify the symptom of arm hemiparesis/hemiplegia and 92 children (74.8%) were able to answer the question regarding speech disturbances. However, the number of correct answers to the question regarding the appropriate course of action in case of a stroke was the lowest among all the questions (10.6% of participants gave a correct answer). Furthermore gender and age did not play a significant role (*p* = 0.571 and 0.635, respectively).

**Conclusion:** Although more than half of the enrolled preschool children could recognize stroke symptoms before their participation in the educational program, their baseline stroke knowledge, prior to their training, is low. Concurrently, they do not have sufficient knowledge on how to react appropriately in the event of a stroke. Therefore, awareness programs focusing on developing stroke literacy to children are needed, to ensure children will seek urgent medical care in case of a stroke.

## Introduction

It is estimated that one million people suffer a stroke worldwide (1). During a stroke, every minute counts, as the sooner a patient receives medical attention, the better the chance for surviving and preventing disability. Worldwide, stroke is not only the second highest cause of death, but also a leading cause of a chronic disability (2), dementia and depression (3). Strokes can be classified into ischemic or hemorrhagic, with ischemia being responsible for the majority of strokes (4). The successful management of stroke is based on rapid reperfusion of intravenous thrombolysis or endovascular thrombectomy, that can reduce a possible disability, but both of them are time-critical (5). There are restricted “time windows” in which these treatments are most efficient. Thus, reducing the time from stroke to arrival at the hospital is the key to maximize the benefits of these therapies as “time is brain.” Nevertheless, statistics show that, on average, the public still lacks basic stroke knowledge and patients continue to arrive in the emergency not fast enough (6). Pre-hospital delay, due to poor public recognition of stroke symptoms, limits the number of patients suitable for proven therapy and increases the incidence of permanent brain injury (7).

Timely medical care depends on the public's awareness of stroke signs, particularly by family members, friends, and bystanders that are in key position to act fast and call a medical emergency number in time. Children are in key position to witness a stroke as they spend a lot of time with grandparents in various cultures (8), who are in higher risk of suffering a stroke (9). Therefore, children can act as adjustment levers for better stroke outcomes in society (10).

Educational interventions about stroke, such as “Hip Hop Stroke” (10) and “Stroke 112” (11) are estimated to be effective for both children and family members through in-house communication. In this regard, the FAST (Face, Arms, Speech, Time) 112 Heroes educational program (12), is unique in that it addresses preschool children who are still in the process of developing communication and learning skills. Creating automatic knowledge gains at this age, by increasing the recognition of stroke symptoms, will lead to increasing stroke knowledge to the children's parents (13). Even if children won't be in a position to seek medical help for a family member, the knowledge transferred to them and their extended family will be of benefit. This will build their understanding and awareness on how to act appropriately through their own cultural lens and create systematic educational changes that will ultimately affect the wider stroke community.

Many studies have been conducted to evaluate stroke awareness in adult populations (14, 15). However, little is known in the stroke knowledge research about young populations, especially in Greece. In some cultures, children spend a lot of time with people who may be at risk for stroke. For this reason, more data is needed about their stroke knowledge. Therefore, we created a questionnaire to evaluate the stroke preparedness of young children.

Our primary goal is to evaluate preschoolers' knowledge levels on stroke symptomatology and the appropriate course of action in case of a stroke before their participation in the educational program FAST 112 Heroes. Our secondary goal is to inspire researchers to design more educational interventions, in Greece, that will educate children on stroke signs and teach them the adequate chain of actions in the event of witnessing a stroke.

## Materials and Methods

**Figure 1:**
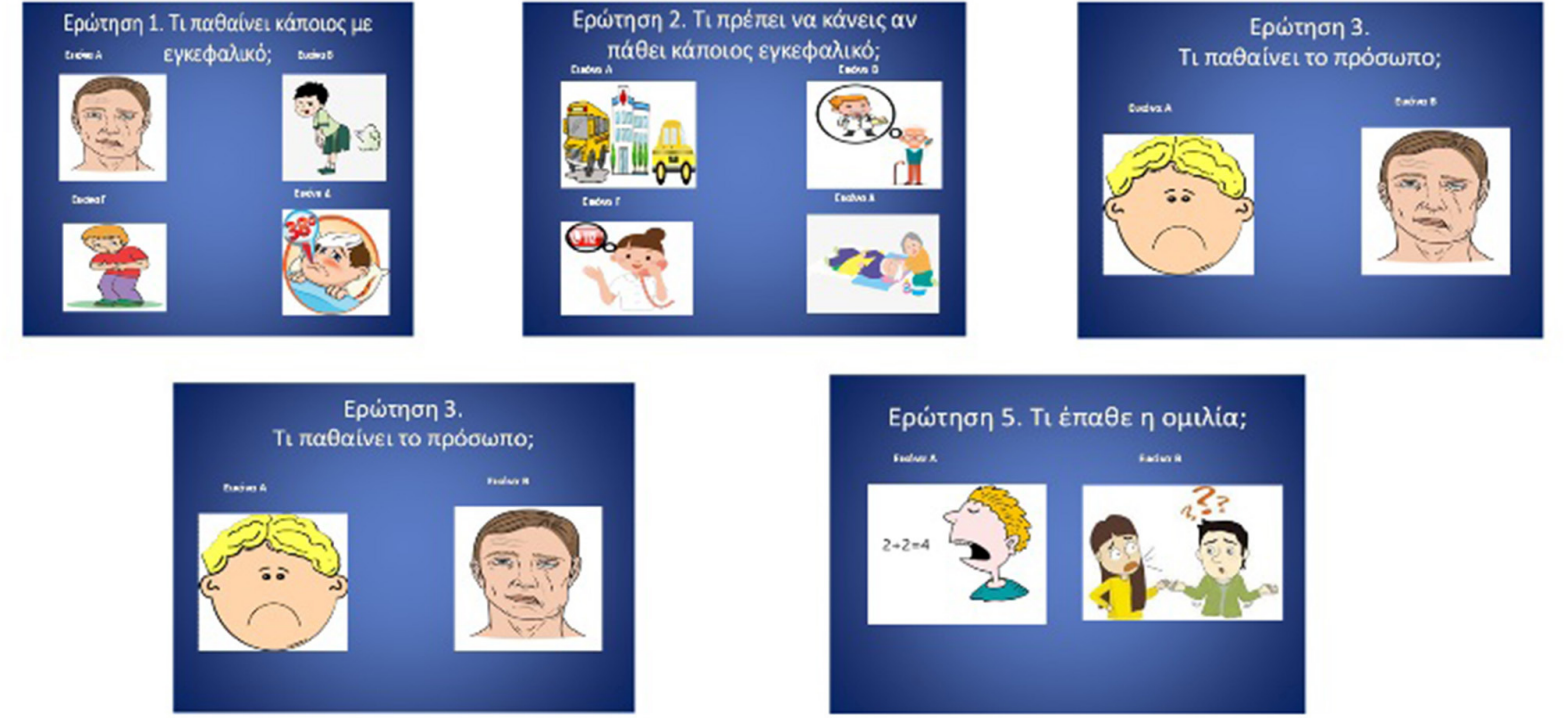
Picture-based stroke literacy test, age-adjusted for young children.

Traditional questionnaires involved examining baseline stroke knowledge (16), which was translated and modified in Greek by bilingual personnel from the Department of Educational and Social Policy (with two-fold back translation). In brief, all images were used as stimuli and contained five age-appropriate, multiple choice questions in animated pictures with headings in the Greek language. To account for the fact that most kindergarten children cannot read, the questionnaire had five multiple choice questions that featured animated pictures and a verbal explanation, provided by the investigator, a technique that has been proven to be age-appropriate for preschoolers (17). The two first questions contained four possible answers while the other three questions contained two possible answers. Each question had only one correct answer. The questionnaire items and the verbal explanations are shown in Figure 1 and Table 1, respectively.

**Table 1:** Verbal explanations provided for each question of the test.

**Question 1**: What is happening when someone is having a stroke?	**Picture A**: One side of the face droops.	**Picture B**: They have gas.	**Picture C**: Their stomach hurts.	**Picture D**: They have fever.
**Question 2**: What is the appropriate course of act in case of a stroke?	**Picture A**: You should hire a taxi/take the bus and take the patient to the hospital.	**Picture B**: You should call the doctor.	**Picture C**: You should call 112.	**Picture D**: You should take the patient to bed.
**Question 3**: What happens to the face after a stroke?	**Picture A**: The face droops from both sides.	**Picture B**: The face droops from 1 side.	–	–
**Question 4**: What happens to the arm after a stroke?	**Picture A**: The arm is weak or numb.	**Picture B**: The arm is in plaster because it is broken.	–	–
**Question 5**: What happens to speech/ after a stroke?	**Picture A**: Everything is fine, they can even do math.	**Picture B**: Their speech is slurred or garbled.	–	–

In order to check the validity of the questionnaire, we conducted a focus group with members of the “Super Grand League Team,” a team of professionals that involves kindergarten teachers, speech language pathologists, psychologists, and special education teachers (12). The focus group unanimously agreed regarding the content of the questions. Minor modifications were suggested, for example removing a question regarding the FAST acronym (i.e., “What does each letter of the F.A.S.T. mnemonic represent?”).

Before the children's knowledge assessments, both information sheets and consent forms were handed out to the participants' parents, who all gave their permission for their children to participate in the study. The questionnaire was administered individually to each child, before the implementation of the program, in a quiet classroom, on a school day, without distractions. The session began with some introductory questions, such as “What is your name,” “How old are you,” and “Have you ever heard the term ‘stroke’?” All responses were verbally collected as well as written down by the examiner. The mean time for the completion of the questionnaire was 5 min.

### Participants

Of the 137 children recruited from the Northern Greece cities of Thessaloniki and Alexandroupolis, 123 children (65 boys, 58 girls, aged 4–6.5 years; mean age: 5.3, S.D.: 0.59), participated in the study. Exclusion criteria included special needs and other neurological difficulties. All children were kindergarten students, attending public schools. Some age categories (e.g., 4- and 4.5-years- old) included only a few children, leading to difficulties in analysis and inferences. Thus, we decided to collapse the data into two main groups (4.0–5.9 and 6.0–6.5 years) based on the number of children in each group. The first group (i.e., 4–5.9 years old) included 42 children whereas the second group (i.e., 6–6.5 years old) included 81 children.

## Results

The present study set out to answer two questions, both concerned with baseline knowledge of stroke symptomatology and stroke preparedness in young children. First, we aimed to explore the baseline knowledge that children of ages 4–6.5 years have. Second, to explore whether children can adequately state the actions needed for appropriate response in case of a stroke.

**Figure 2:**
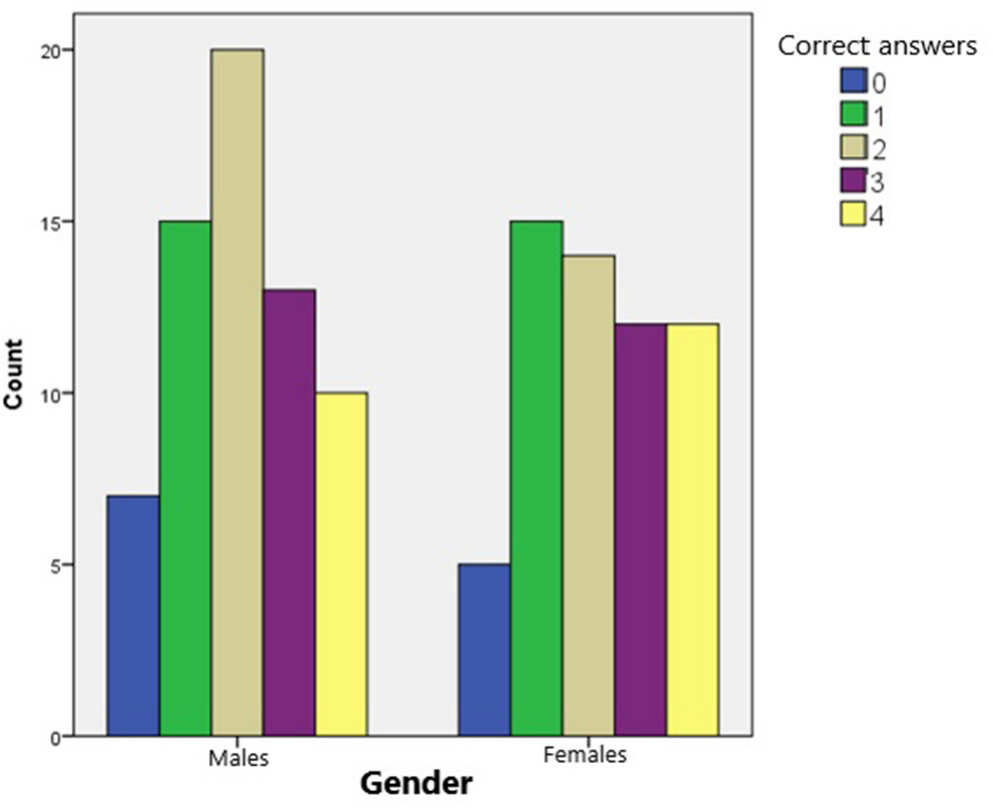
Number of children who answered correctly, based on their gender (Note: minimum of correct answers possible: 0; maximum of correct answers possible: 4).

**Figure 3:**
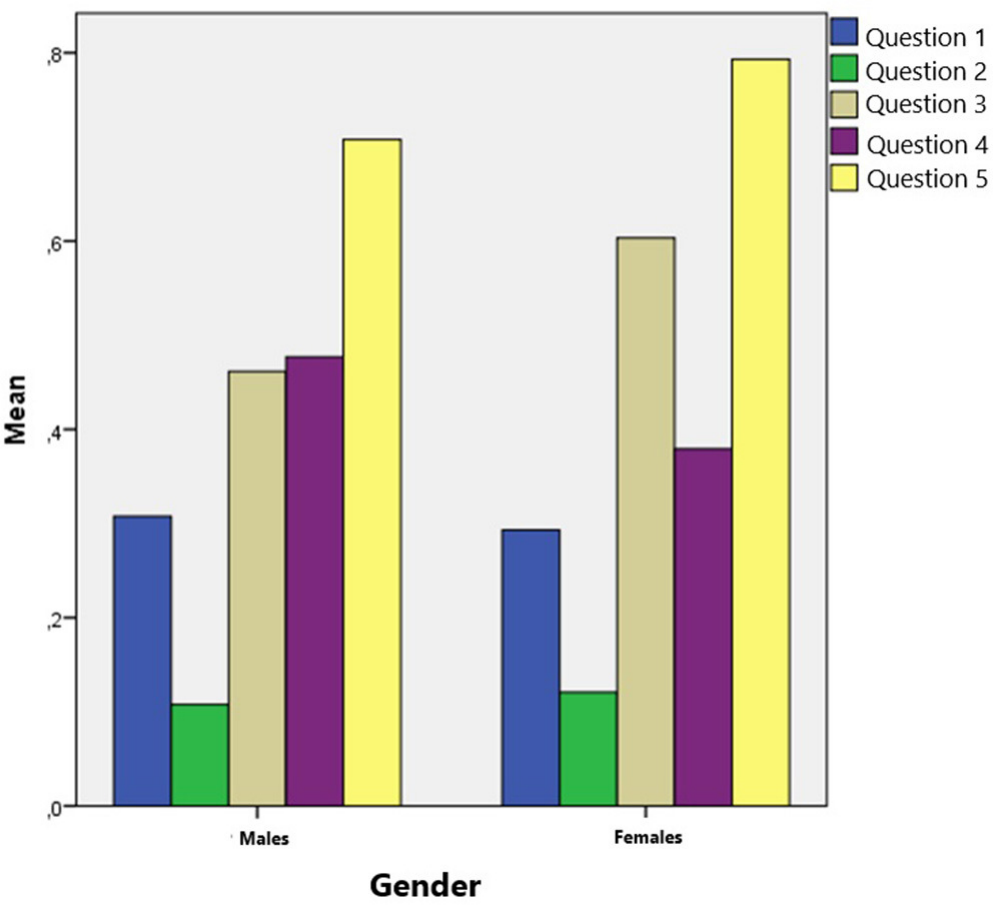
Mean score of children's correct answers to each question of the test, as compared to their gender (Note: 0 is for wrong answers; 1 is for correct answers).

As illustrated in Figure 2, the number of correct answers in males appears as an approximately normal distribution. Most of the boys answered two questions correctly while the extreme cases (0 or 4 correct answers) representing the smallest portion of males. Most of the females' answers did not follow a normal distribution. None of the participants answered all questions correctly. However, both females and males scored better in the questions regarding the stroke symptoms. As can be seen in Figure 3, females scored slightly better to the questions about stroke symptoms. Gender did not play a significant role in children's answer (*t* = 4.265, *p* = 0.571).

**Figure 4:**
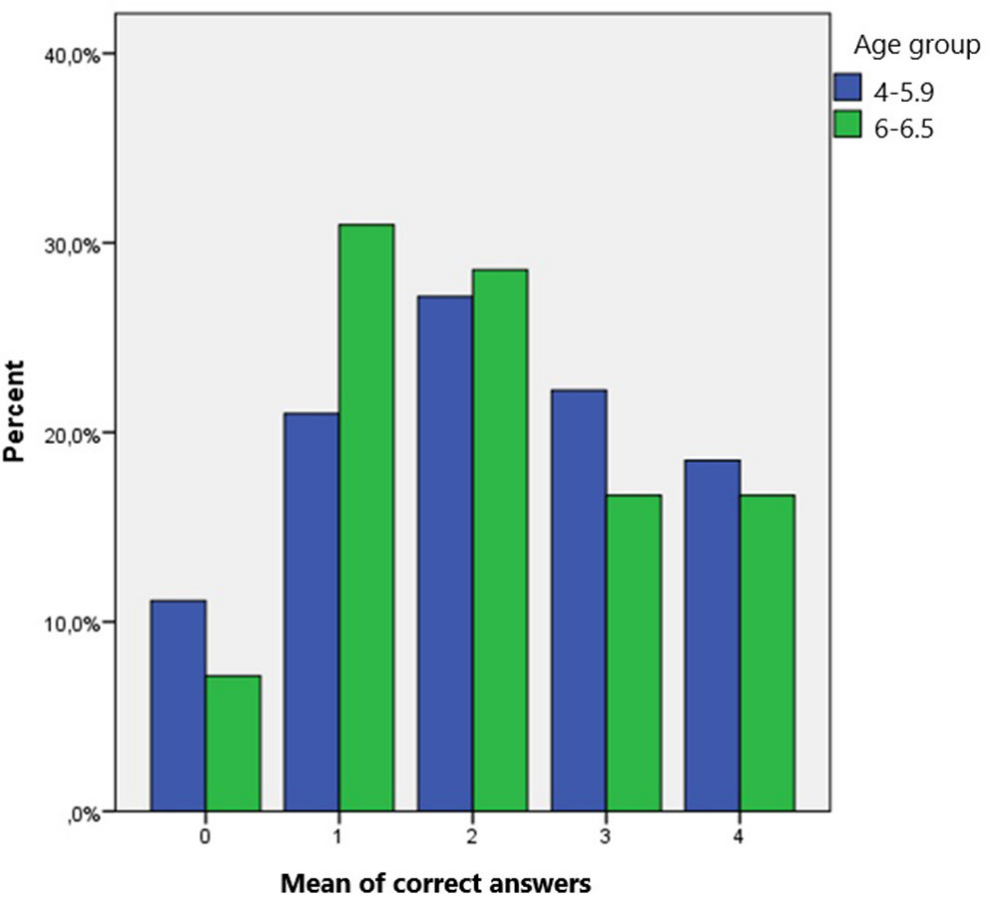
Mean score of correct answers given, based on children's age group.

Age did not play a significant role in children's answers (*t* = 0.467, *p* = 0.635), as there were no significant differences in the percentage of correct answers depending on age. Children under 6 years old gave slightly more correct answers to questions regarding the stroke symptoms (Figure 4). Children under 6 years old answered correctly 43.2% of the questions while older than 6 years old answered correctly 40.95% of the questions (Table 2). Both groups of participants scored better in the questions regarding the stroke symptoms. The mean number of the correct answers given in regard to the stroke symptoms was almost the same depending on the two age groups. Children from the younger group answered questions 1 and 4 correctly while children from the older group answered questions 2, 3, and 5 correctly.

**Table 2:** The distribution of correct answers for each age group.

**Group ages**	***N***	**% of correct answers**	**Standard deviation**
4–5.9	81	43.2099	25.38834
6–6.5	42	40.9524	24.17513
Total	123	42.4390	24.90446

Preliminary analysis evaluating the questionnaire, indicated that 37 children (30.1%) could recognize a stroke (question 1); 13 children (10.6%) were able to answer the question regarding the appropriate course of action (question 2); 65 (52.8%) could spot a stroke by face (question 3); 53 children (43.1%) were able to answer the question regarding the arm symptom in a stroke (question 4); 92 children (74.8%) were able to answer the question regarding the speech in a stroke (question 5). Question 2 was the most difficult for the participants to answer correctly while Question 5 was the easiest for the participants to answer correctly.

## Discussion

Our prediction that children do not have sufficient baseline knowledge is in line with previous literature arguing that children lack of basic stroke literacy (16, 18). The majority of children in this work could not identify the appropriate course of action in case of a stroke and no child completed the test with a perfect score. Recent studies have shown that educational programs can have a positive impact, not only on children's stroke knowledge, but also on their extended families, since children can be leveraged as conduits and transfer stroke literacy to the family members (19–22). We show that young children are not aware enough of the stroke symptomatology and thus suggest more school-based interventions that will deliver stroke knowledge to children. The results revealed that children were able to recognize the stroke symptoms after the intervention, and maintained the knowledge gained for almost a month later.

Gaining such knowledge helps children build resiliency skills since they become prepared for an unfortunate event that may happen in a family. It is very beneficial for children to become acquainted with symptoms of disorders so that they normalize in their minds these events if or when they happen (23). It is commonly accepted, and experienced, that the majority of children adjust well to unfortunate events and inconveniences, and thus, they can act as supportive units in the management of a stroke event involving a family member (24).

We summarize the major findings of this work by pointing out some limitations. First of all, sample size of young children was small. Nevertheless, we have tried to offer some speculations and can only hope that our investigation will stimulate further research on measuring baseline stroke knowledge for young children. In our interpretation of stroke knowledge questionnaires, the number of answers differed in questions 3, 4, and 5. The fact that questions regarding stroke symptoms had only two possible choices might be the reason why most children answered slightly better in questions 3, 4, and 5, which addressed face drooping, arm weakness and speaking trouble symptoms as stroke symptoms. The reason we chose this number of possible choices is justified by Alloway et al. (25) who suggest that the visuospatial working memory measures of young children demand parallel processing and storage of information, an association which gradually increases from the age 4–6 to older ages. Of course, more questions with the same number of possible answers per question could increase the test reliability.

The most interesting result in our questionnaire was that most of the participants struggled to find the correct answer to Question 2, concerning the appropriate way to act in case of a stroke. This is in accordance with the literature (26, 27) that points to the fact that young children generally do not know much about stroke. There are limited local campaigns or educational interventions to increase stroke knowledge as well as the appropriate emergency number in case of a stroke. What we hope to have achieved, even with the small sample tested here, is to have sparked educators and researchers' attention in encouraging people to further investigate stroke knowledge in young children and highlight the necessity of training emergency responses.

The present study focused on the baseline knowledge of children of stroke symptoms and immediate reaction to those on their behalf. One suggestion for future studies could be to extend the investigation of young children's involvement or engagement with family members that suffer from a stroke in the long run. How do they react behaviorally and psychologically to these family members? Do they assist in their daily care? Do they become attached or remoted?

It is increasingly important that local state agencies will systematically offer such informative sessions with public and private schools so that knowledge is repeated in children's minds and thus mastered. Usually, such actions are conducted only in training for emergency situations but should be integrated in the school curriculum, as part of a national educational program.

## Data Availability Statement

The raw data supporting the conclusions of this article will be made available by the authors, without undue reservation.

## Ethics Statement

Study approval was obtained from the Committee for Research Ethics of the University of Macedonia (Thessaloniki, Greece) (14/15.06.2020). Written informed consent to participate in this study was provided by the participants' legal guardian/next of kin.

## Author Contributions

All authors listed have made a substantial, direct and intellectual contribution to the work, and approved it for publication.

## Conflict of Interest

The authors declare that the research was conducted in the absence of any commercial or financial relationships that could be construed as a potential conflict of interest.
